# Athlete availability and incidence of overuse injuries over an athletics season in a cohort of elite Swedish athletics athletes - a prospective study

**DOI:** 10.1186/s40621-020-00239-0

**Published:** 2020-05-04

**Authors:** Andreas Lundberg Zachrisson, Andreas Ivarsson, Pia Desai, Jon Karlsson, Stefan Grau

**Affiliations:** 1 Center for Health and Performance, Department of Food and Nutrition, and Sport Science, University of Gothenburg, Box 300, 405 30 Gothenburg, Sweden; 2 School of Health and Welfare, Halmstad University, Kristian IV:s väg 3, 301 18 Halmstad, Sweden; 3 Department of Orthopaedics at Institute of Clinical Sciences, Sahlgrenska Academy, University of Gothenburg, Göteborgsvägen 31, 431 80 Mölndal, Sweden

**Keywords:** Overuse injuries, Elite athletics, Athlete availability, Incidence rate, Elite athletes

## Abstract

**Background:**

Athletics is a sport with a high incidence of injury, where most injuries are caused by overuse. Research on injury incidence and the occurrence of overuse injuries during a season in athletics is scarce. An athlete availability (unrestricted ability to participate in training or competition) of less than 80% has been linked with athletes being less likely to reach their performance goals. The purpose of this study was to estimate the monthly injury incidence rates, athlete availability, and the overuse injury incidence rate per 1000 athletics-hours of training in a cohort of Swedish elite athletics athletes.

**Methods:**

The cohort consisted of 59 male and female elite athletes competing in either middle or long-distance running, sprint, or jumping events. Injury and training data were collected during one athletics season, from October to the end of August. All injury data were collected by medical professionals. Training data were collected monthly, and consisted of event-specific training diaries covering training sessions, training days, and non-training or non-competition days. Monthly injury incidence rates were based on the number of new injuries per month in relation to the number of exposed (injury-free) athletes.

**Results:**

The overall injury incidence rate for all athletes was highest in October (22.0%). Monthly injury incidence rate for middle and long-distance runners was highest in October (26.1%), for sprinters in April (19.0%), and for jumpers in October (21.4%). The overall athlete availability was 78.0% for the cohort. Sprinters had the lowest athlete availability (71.4%), followed by jumpers (77.3%), and middle-distance and long-distance runners (82.7%). Female athletes (76.5%) had a lower athlete availability than male athletes (79.7%). The injury incidence rate was 1.81 injuries per 1000 athletics hours of training. Middle and long-distance runners had the highest injury incidence rate (2.38), followed by jumpers (1.62), and sprinters (1.34).

**Conclusion:**

Monthly injury incidence rates during a season appears to correspond to periods of high training volume (conditioning phases and training camps). The low overall athlete availability (> 80%) indicates that many Swedish elite athletes are less likely to reach their full potential.

## Background

Athletics (track and field) is a global sport with over 200 member nations in the World Athletics organization (About World Athletics n.d.). Major international championships are arranged every other year, and athletics is the largest sport at the Olympic summer games in terms of number of athletes competing in different events (sprint, middle and long distance running, jumping, throwing, and combined events) (Engebretsen et al. 2013). Athletics is characterized as a sport with high training demands (Ahuja and Ghosh 1985; Jacobsson et al. 2013). Top-level athletes competing in athletics are subject to a high risk of injury, which has been established in numerous studies (Ahuja and Ghosh 1985; Jacobsson et al. 2013; Alonso et al. 2009; Alonso et al. 2012; Opar et al. 2015; Jacobsson et al. 2012; D'souza 1994; Watson 1987; Bennell and Crossley 1996; Lysholm and Wiklander 1987; Edouard and Alonso 2013). A consensus statement for athletics describing a standardized method of collecting injury data was published in 2014 to attain more reliable and comparable evidence for epidemiological research (Timpka et al. 2014). The consensus statement was applied with regard to injury severity and onset of injury. Injury definition was slightly adapted by adding medical professionals to gather injury diagnosis instead of athlete self-reporting. Further, only injuries were considered that had an impact on athletics training or competition.

The majority of all injuries in athletics can be classified as overuse injuries (OI) defined as “a condition to which no identifiable single external transfer of energy can be associated. Multiple accumulative bouts of energy transfer could result in this kind of injury “(Timpka et al. 2014), and most OI affect the lower extremities (Jacobsson et al. 2013; Bennell and Crossley 1996; Lysholm and Wiklander 1987; Edouard and Alonso 2013). Research on monthly injury incidence rates in athletics is still scarce. Only a few studies have reported data related to the timing of injury onset during an athletics season. No information on potential influencing factors, such as type and quality of training or event group (e.g. sprint or middle and long-distance runners), has been presented in conjunction with the timing of injury (Jacobsson et al. 2013; D'souza 1994; Lysholm and Wiklander 1987). There appears to be no differences in overall injury incidence rates between event groups, but this remains unclear due to different study designs (e.g. retrospective vs. prospective), injury definitions (e.g. injuries affecting training or not), and sample (sub-elite vs. elite) used. There are conflicting results about injury incidence rates per 1000 athletic hours of training with regard to athlete gender (Ahuja and Ghosh 1985; Jacobsson et al. 2013; D'souza 1994; Watson 1987; Bennell and Crossley 1996). Jacobsson et al. showed that a high training load (intensity and volume) increases the risk of injury in elite athletics, although only used a relative measure combining training hours and intensity (training load rank index) prior to the start of the study (Jacobsson et al. 2013). Athlete availability (unrestricted ability of athletes to participate in training or competition) has also been shown to be important, athletes with less than 80% athlete availability during a season were less likely to reach their performance goals than athletes who had a higher athlete availability (Raysmith and Drew 2016). The same pattern has also been seen in youth athletics athletes (Watson 1987).

Therefore, the aim of this study was to estimate the monthly injury incidence rates, the overall and individual athlete availability, and the injury incidence rate of OI per 1000 athletic hours of training in a cohort of Swedish elite athletics athletes from three event groups.

## Methods

### Study population

Inclusion criteria was that all athletes placed in the top six of the Swedish national championship or top three of the Swedish youth national championship. Furthermore, all athletes had to be registered with an athletics club in Gothenburg, be at least 18 years of age, and have no musculoskeletal pain or injury affecting their performance as confirmed by the study’s physiotherapist at enrollment. The Gothenburg elite athletics athletes’ cohort represents approximately 22% of the total Swedish elite athletics cohort (Johan Wettergren, Sweden Athletics and GFIF, personal communication).

**Fig. 1:**
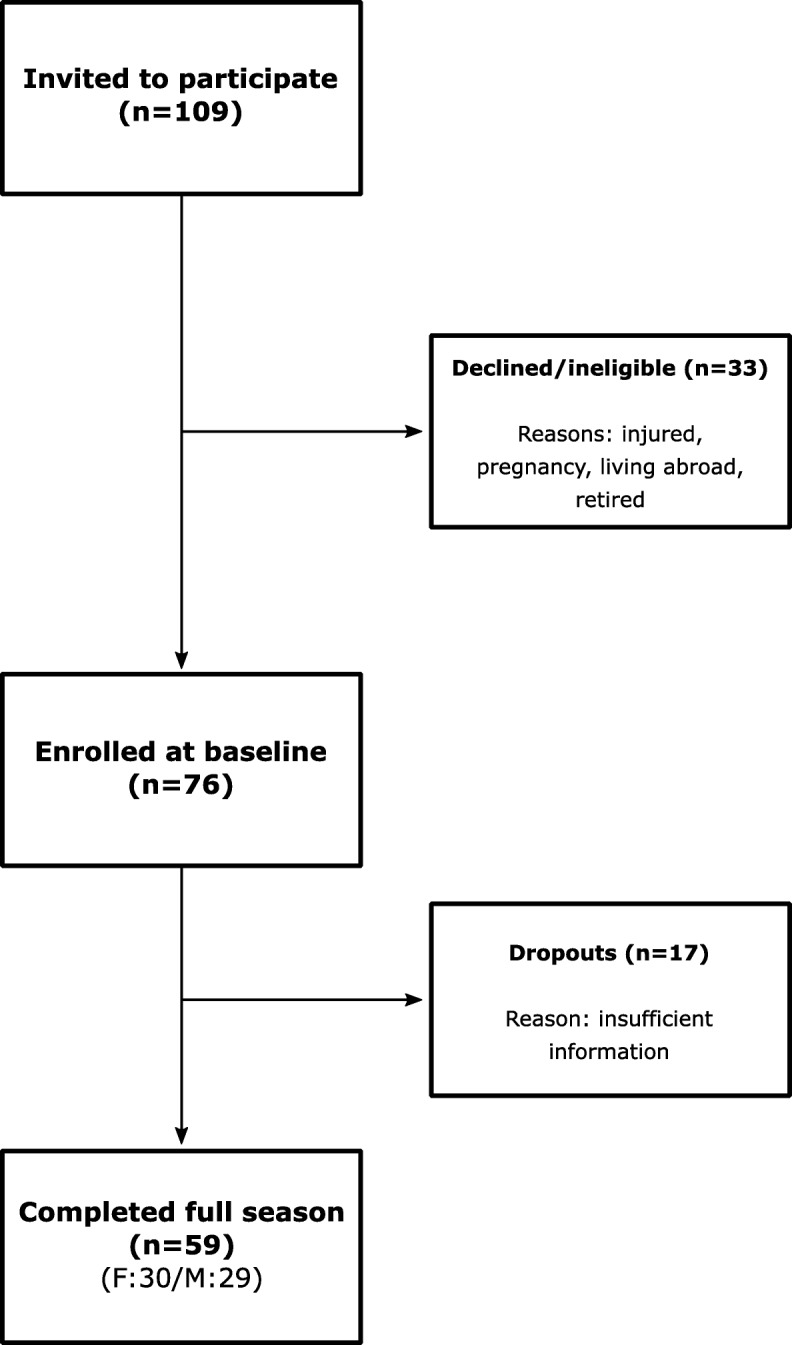
Flowchart of athlete enrollment

The Gothenburg Athletics Association (GFIF) assisted with recruitment by compiling a list of athletes based on the inclusion criteria. The study leader contacted all athletes on the list and invited them to join the study. Both male and female athletes were recruited from three event groups: middle and long-distance runners (800 m and upwards), sprinters (60 m to 400 m, including hurdles), and jumpers (long jump, triple jump, high jump, and pole vault). An initial number of 109 athletes were invited to take part in the study. Thirty-tree declined to participate or were found to be ineligible. During the season, 17 athletes were considered dropouts and not included in the final study population, as they did not submit a complete set of training diaries during the data collection period. The final study population consisted of 23 middle-distance and long-distance runners, 23 sprinters, and 13 jumpers (*n* = 59) (Fig. 1) (Table 1.).

**Table 1:** Overall study population

	**Overall (*n*** **= 59)**	**Females (*n*** **= 30)**	**Males (*n*** **= 29)**
**Age (years)**	21.6 (2.8)	22.2 (3.3)	21.1 (2.0)
**Height (m)**	1.77 (0.08)	1.71 (0.05)	1.85 (0.05)
**Weight (kg)**	67.2 (9.3)	60.6 (6.5)	74.1 (6.4)
**BMI (kg/m**^**2**^**)**	21.2 (1.7)	20.7 (1.7)	21.7 (1.6)
**Weekly average training hours**	14.0 (3.4)	13.7 (3.0)	14.4 (3.7)
**M/L (n)**	23	13	10
**Sprint (n)**	23	8	15
**Jump (n)**	13	9	4

Mean values for age, height, weight, BMI, and weekly training hours

Standard deviation in parentheses. *M/L* middle and long-distance runners, *n* number

### Study design

The present study is a prospective cohort study conducted during one Swedish athletics season. Participating athletes were enrolled starting in October. All athletes had to complete one full season of athletics, from the first day of October until the end of August the following year, which was a total of 335 days. After consulting the elite coaches who represented the participating athletes, the month of September was excluded from data collection for all athletes from all event groups, as most athletes take time off to rest during this phase in preparation for the next season.

The season was split into four different phases: conditioning phase one (October through December), indoor competition (January through February), conditioning phase two (March through May), and outdoor competition (June through August). The phase durations were determined together with the responsible elite coaches. The present study is the second part of a previously published study protocol (Zachrisson et al. 2018). All participants signed an informed written consent form to partake in the study. Participants had time to ask questions regarding their participation and could withdraw their participation in the study at any time. All athletes were given a coded ID (identification) number to ensure anonymity. The study was approved by the Regional Ethical Committee in Gothenburg (dnr. 723–16), and follows the STROBE statement (Vandenbroucke et al. 2007).

### Training data collection

All athletes submitted monthly training diaries during the season. The training diaries were developed together with the athletes’ coaches and each event group had their own specific version. However, all training diaries used the same basic design for comparability. The training diary consisted of an Excel sheet with information to be filled in daily (Zachrisson et al. 2018). At the end of each month, the athlete or the coach submitted the diary to the study leader via e-mail. The information collected consisted of the number of training sessions (including all competitions), training days (including all competitions), and non-training or non-competition days. Non-training or non-competition days were defined as days the athletes were ill, conducted alternative training or rehabilitation (i.e. not athletics training), or no athletics training at all. At the end of the study, all athletes provided the study leader with the average number of weekly training hours they had during the past season.

### Injury definition

An injury was defined as any musculoskeletal pain felt during athletics training or competition that inflicted a non-voluntary reduction of or complete stop from athletics training for at least 24 h, and was diagnosed by a trained medical professional, e.g. a physiotherapist and/or sports physician (Zachrisson et al. 2018).

### Injury data collection

All athletes could directly contact the study leader by e-mail or telephone to schedule an appointment for a physical examination by the study’s medical professional. Each athlete could also report an injury through a mobile phone application developed for the study, or by noting it in their training diary. The mobile phone application consisted of questions regarding pain during training or competition. If any pain was reported in the mobile application or in the training diary (both of which were reviewed by the study leader daily), the study leader immediately contacted the afflicted athlete to make an appointment for a physical examination.

The medical professionals, consisting of the study’s physiotherapist and orthopedic surgeon, offered examinations free of charge to the athletes throughout the study. If the athlete was treated by an external medical professional, the injury information was collected from the external medical professional by e-mail, phone, or in person.

A standardized injury report form was used by the study’s medical professionals who examined and treated the athletes. All injuries were classified according to their onset as sudden onset due to overuse, or gradual onset due to overuse (Timpka et al. 2014). Recurring, traumatic or acute injuries were diagnosed and documented by the medical professionals, but not included for analysis. Only new injuries classified with an onset linked to overuse were included for analysis. The first day of injury was noted when the athlete first reported pain that led to a non-voluntary reduction or cessation of athletics training. An injured athlete was considered injury-free when reporting full return to athletics training (i.e. no changes from their normal training) in their training documentation. To be included in the analysis, all OI had to be diagnosed as new injuries by the medical professionals. All OI were recorded by the study’s physiotherapist.

### Statistical analyses

Mean and SD were used to display the study population. Athletes were divided according to the three event groups; middle and long-distance running (800 m and upwards), sprint (60 m up to 400 m including all hurdle event), and jumping (long jump, triple jump, high jump, and pole vault).

Overall injury proportion was calculated by dividing the number of injured athletes by the number of athletes (total cohort) during the 11 month period.

To calculate the monthly injury incidence rate in each event group, the correct value for the total number of exposed athletes for each month had to be quantified first. An exposed athlete was defined as an athlete that was injury-free and could participate in training or competition without restrictions (Knowles et al. 2006). To quantify the exact amount of exposed athletes, the athletes with carry-over injuries from the last month into the current month (e.g. 10 days or 17 days) had to be quantified, as they were then also exposed athletes in the current month but not for the whole time period. Therefore, the number of injury days of the current month of the carry-over athletes was divided by the number of days for that month (=number of un-exposed athletes per month and event group). This value was then subtracted from the total number of athletes for each event group (=number of exposed athletes). The number of new injuries for each month and event group was then divided by the number of exposed athletes per event group to estimate the monthly injury incidence rates in percentage (%). An exception was made for the month of October (start of study), as there were no carry-over injury days from September. For October, the number of new injuries was divided by the number of exposed athletes per event group.

### Calculation example of monthly injury incidence rates

If the carry-over injury days from December into January was 31 days, then this value was divided by the number of days in January (31/31). This value would represent one fully un-exposed athlete for January. Then this value was subtracted from the total number of athletes in that event group, (e.g. 23–1) to estimate the number of athletes that were exposed (*n* = 22) to athletics training or competition during January. If the cohort of athletes suffered from two new injuries (in total) in December, then the monthly injury incidence rate would have been 2/22 which is 9.1%.

To calculate the average number of monthly training sessions for each event group, the total number of training sessions was divided by the number of exposed athletes.

The following procedure was used to calculate athlete availability: First, the total number of healthy days for each athlete was converted to healthy weeks (7 days is 1 week). In a second step, the length of the study (47.5 weeks) was divided by 100 and then multiplied by the number of healthy weeks (athlete availability in %). Athlete availability was calculated for each athlete individually as well as for the overall cohort, gender, and event group. This was a modification of previous models to determine incidence and severity (Raysmith and Drew 2016; Bahr et al. 2018).

The injury incidence rate per 1000 athletic-hours of training was calculated by dividing the number of injuries per athlete by the yearly athletic hours of training, and then multiplying by 1000 (Phillips 2000).

All data were analyzed using SPSS statistics (Version 25, IBM Inc., Armonk, New York).

## Results

This study examined the data of 59 Swedish elite athletes competing in athletics who completed a full season. All athletes completed the most training sessions in October (1719) followed by April (1687) (Table 2). Middle and long-distance runners had the highest average number of training sessions over the season, followed by sprinters and jumpers. Overall, most OI occurred in October (13). Middle and long-distance runners suffered most OI in October (6), sprinters in October (4), December (4), and April (4), and jumpers in October (3) and December (3). Overall, most training sessions and OI occurred in the first conditioning phase from October through December.

**Table 2:** Total and average monthly training sessions of injuries during an athletics season

	**Oct.**	**Nov.**	**Dec.**	**Jan.**	**Feb.**	**March**	**April**	**May**	**June**	**July**	**Aug.**
**Total sessions (overall)**	1719	1641	1583	1528	1222	1520	1687	1526	1337	1436	1247
**Avg. sessions (M/L)**	31.57 (6.8)	35.12 (6.7)	33.64 (8.7)	37.24 (7.0)	29.43 (7.5)	31.96 (7.9)	34.82 (7.3)	33.25 (8.2)	35.83 (7.8)	37.90 (8.0)	32.67 (10.4)
**Avg. sessions (Sprint)**	30.91 (7.4)	30.61 (8.8)	26.39 (8.2)	26.50 (6.5)	20.76 (2.9)	28.97 (3.9)	28.90 (4.4)	27.14 (3.3)	21.63 (2.5)	26.28 (2.6)	23.58 (4.5)
**Avg. sessions (Jump)**	21.69 (5.1)	24.10 (8.8)	24.34 (7.7)	21.94 (6.0)	19.65 (5.2)	18.95 (4.6)	24.21 (3.9)	19.77 (3.9)	19.11 (6.0)	19.25 (6.2)	17.58 (6.1)
**Injuries (overall)**	13	1	9	3	4	6	8	6	7	2	7
**Injuries (M/L)**	6	0	2	0	2	3	4	5	4	1	3
**Injuries (Sprint)**	4	0	4	2	1	2	4	0	2	1	2
**Injuries (Jump)**	3	1	3	1	1	1	0	1	1	0	2

Standard deviation in parentheses. *M/L* middle and long-distance runners

### Monthly injury incidence rates

**Fig. 2:**
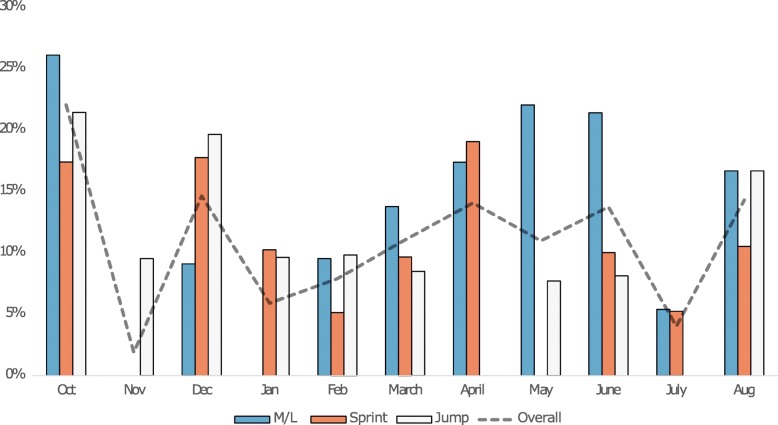
Overall and event-specific monthly injury incidence rate (%). M/L = middle and long-distance runners

**Fig. 3:**
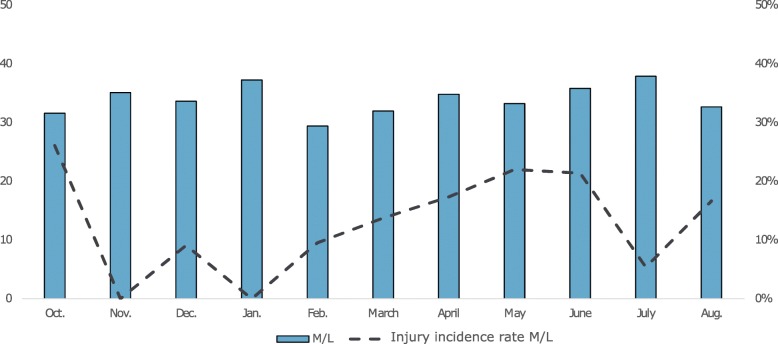
Monthly average number of training sessions (y-axis, left) and monthly injury incidence (%) (y-axis, right). M/L = middle and long-distance runners

**Fig. 4:**
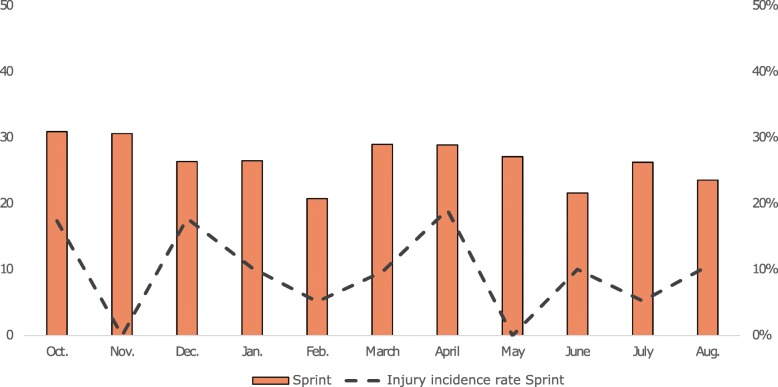
Monthly average number of training sessions (y-axis, left) and monthly injury incidence (%) (y-axis, right)

**Fig. 5:**
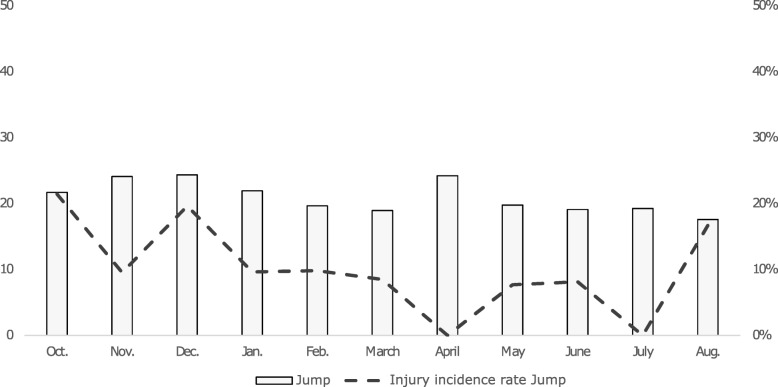
Monthly average number of training sessions (y-axis, left) and monthly injury incidence (%) (y-axis, right)

The overall injury incidence rate was highest in October (22.0%), followed by December (14.6%) (Fig. 2). The monthly injury incidence rate for middle and long-distance runners was high in October (26.1%) and May/June (22.0 - 21.4%), with January being the month in which most training sessions were conducted (Fig. 3). Sprinters had a high injury incidence rate in December (17.7%) and April (19.0%), whereas most of their training sessions were conducted in October (Fig. 4). Jumpers had the highest injury incidence rate in October (21.4%) and December (19.6%), and December is the month they conducted the highest number of training sessions (Fig. 5).

### Athlete availability

**Fig. 6:**
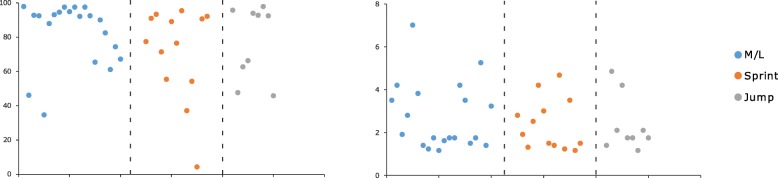
Distribution of athlete availability (left, %) and incidence (right, n/1000 h) at an individual level. M/L = middle and long-distance runners

Athlete availability was 78.0% (CI: 71.14–84.91) for the overall study population. Sprinters had the lowest athlete availability (71.4%, CI: 55.02–87.83), followed by jumpers (77.3%, CI: 60.71–93.84) and middle and long-distance runners (82.7%, CI: 74.02–91.28). Female athletes had a lower athlete availability (76.5%, CI: 65.73–87.30) than male athletes (79.7%, CI: 70.40–88.97) (Table 3). There was a large individual variation of athlete availability in all event groups (Fig. 6).

**Table 3:** Overuse injuries, injury proportion, and incidence during an athletics season

	**All**	**Male**	**Female**	**M/L**	**Sprint**	**Jump**
**Overuse injuries**	66	33	33	30	23	13
**Injured athletes**	42 (71%)	20 (67%)	22 (76%)	20 (87%)	13 (57%)	9 (69%)
**n/1000 h**	1.81 (1.39–2.23)	1.79 (1.13–2.45)	1.83 (1.29–2.37)	2.38 (1.67–3.09)	1.34 (0.73–1.95)	1.62 (0.78–2.46)
**Athlete availability (%)**	78.0 (71.14–84.91)	79.7 (70.40–88.97)	76.5 (65.73–87.30)	82.7 (74.02–91.28)	71.4 (55.02–87.83)	77.3 (60.71–93.84)

For incidence rate and athlete availability 95% CI in parentheses. *M/L* middle and long-distance runners

### Injury incidence rate per 1000 athletic hours of training

Overall, the athletes suffered from 66 OI equally distributed between genders. Middle and long-distance runners had the highest proportion of injured athletes (87%) and suffered the most OI (30) (Table 3). The injury incidence rate per 1000 athletic hours of training in the study population was 1.81. Middle and long-distance runners had the highest injury incidence rate (2.38), followed by athletes competing in jumping events (1.62), and sprint (1.34). Female athletes had a slightly higher injury incidence rate (1.83) than male athletes (1.79) (Table 3). There was a large variation of injury incidence rate per 1000 athletic-hours of training at an individual level in all event groups (Fig. 6).

## Discussion

The aim of this paper was to estimate monthly injury incidence rates, athlete availability (athlete’s unrestricted ability to participate in training or competition), and injury incidence rate of OI calculated per 1000 athletic hours of training in a cohort of Swedish elite athletics athletes from three event groups. The three event groups consisted of middle and long-distance runners, sprinters, and jumpers.

In the present study, most OI occurred in autumn closely followed by spring (the two conditioning phases). This is in contrast to Jacobsson et al. (Jacobsson et al. 2013), who reported most of injuries in April (the beginning of their study), and a continuous decrease until the end of their study 1 year later. The differences may be due to the self-reporting of injuries in Jacobsson et al. (Jacobsson et al. 2013), with over-reporting at the beginning of the study and continuous under-reporting towards the end of the study, as well as the use of a different injury definitions. Another explanation could be that the athletes in our cohort rested for the entire month of September and then increased training volume and/or training intensity too quickly (Ballas et al. 1997).

Lysholm et al. (Lysholm and Wiklander 1987), who studied sprinters and marathon runners, found most injuries in sprinters in March and July, which is in contrast to the present cohort of sprinters who reported most OI in October, December, and April. A possible explanation could be that the high elite level sprinters in the present study tried to hit peak form before the indoor competitions which led to an increased number of OI compared to district level athletes in Lysholm’s study. Moreover, the high elite level sprinters in the present study possibly sustained OI during training camps in the final preparation phase before outdoor competitions. The long-distance/marathon runners in Lysholm’s study (Lysholm and Wiklander 1987) reported most injury days in March, May, and July in comparison to our middle and long-distance runners, who had most injury days in October, May, and June. This could be explained by the different types of runners (long distance/marathon street runners compared to middle and long-distance track runners) and their different training periodization during the season. The main competition period for long-distance/marathon runners is spring compared with January/February and during the summer for middle and long-distance track runners.

The higher proportion of OI in middle and long-distance runners (87%) compared to sprinters (57%) and jumpers (69%) could be explained by the greater amount of training sessions over the season. Injury incidence rates for the three event groups differed slightly. For middle and long-distance runners and sprinters, the peak injury incidence rate occurred during the second conditioning phase, which could be correlated to training camps where the athletes have increased training sessions and intensities. The athletes attend these training camps before the outdoor competition starts (training camps for sprinters are in April, and for middle and long distance runners in May). The peak monthly injury incidence rate forjJumpers was in October at the beginning of the season, and could be due to the large increase in training load, as athletes rested in September. The relationship between injury incidence rate and training seems to correspond with each other, which is in accordance with Jacobsson et al. and Lysholm et al. (Jacobsson et al. 2013; Lysholm and Wiklander 1987).

In athletics, previous research has found that athlete availability during the season is related to performance, as the likelihood of achieving a performance goal increased seven-fold in athletes that completed > 80% of planned training weeks (Raysmith and Drew 2016). Furthermore, successful athletes had a highly significant lower incidence of injuries and illnesses, and a highly significant lower total season burden of injuries (Raysmith and Drew 2016). The same detrimental impact of performance due to low athlete availability (availability of team members) has been reported for football and basketball (Drew et al. 2017). The reported average athlete availability for each of the three event groups in the current study is just under or over 80%, with a lower athlete availability for female athletes than for male athletes [Table 3]. The uneven distribution of athlete availability in each of the event groups indicates that many of the athletes in our event groups most likely did not reach their full potential during the season [Fig. 6]. This is especially true for sprinters (7 out of 13 sprinters) and jumpers (4 out of 9 jumpers). The relatively low individual athlete availability values in our cohort can be explained by the generally high number of injuries (also multiple OI for the same athlete) and the severity of the OI.

The overall injury incidence rate per 1000 athletics hours of training in our study was low (1.81) compared to previously published results, which range from 2.5 to 5.8 per 1000 athletics hours of training (Jacobsson et al. 2013; Bennell and Crossley 1996; Lysholm and Wiklander 1987). The lower overall injury incidence rate in the present study may be due to the exclusion of recurrent, traumatic, and acute injuries compared to previous studies (Jacobsson et al. 2013; Bennell and Crossley 1996; Lysholm and Wiklander 1987). Injury incidence rate relative to gender was also considerably lower in our study than previously reported by Jacobsson et al. (Jacobsson et al. 2013) and Bennell et al. (Bennell and Crossley 1996). In contrast to previous studies, female athletes had a slightly higher injury incidence rate than male athletes, which could be explained by the high amount of female middle and long-distance runners and jumpers compared to sprinters in our cohort (Jacobsson et al. 2013; Bennell and Crossley 1996).

### Limitations

Except for one study (Lysholm and Wiklander 1987), previous athletics studies have used a full year to record data, meaning that they include an additional 30 days. However, as most elite athletes in Sweden rest in September, the risk of missing additional injury data is low. Even though the study’s medical professionals were very experienced, it is possible that a hamstring strain was documented as an acute injury and not as an OI. If so, the reported injury incidence rate would be even higher. The medical attention definition from the consensus statement (Timpka et al. 2014) could not be fully implemented. As far as possible, the study’s medical professionals tried to examine all injuries. However, multiple medical professionals were necessary in a few certain circumstances (e.g. when the athlete was training or competing abroad), leading to a possible lower inter-rater reliability (Phillips 2000). As the athletes did not consistently report illnesses, rehabilitation/alternative training, and normal rest days in the mobile application, we had to summarize those days into a non-training/non-competition variable. As no information about the intensity or training load of the training sessions was submitted by the athletes or coaches, only limited training data could be analyzed to research possible relationships between training and OI. Another potential limitation of this study is the relatively low sample size due to the geographical recruitment. Finally, we did not record the athletes’ performance during the season, thus we were not able to link athlete availability to performance outcome.

## Conclusion

Middle and long-distance runners have a high proportion of OI that could be linked to the high amount of training during the season compared to sprinters and jumpers. The injury incidence rates during an athletics season corresponds to periods of high training volume (conditioning phases and training camps). The low athlete availability (under or just over 80%) in each respective event group, as well as at an individual level, indicates that many Swedish elite athletes may not be able to reach their full potential. To avoid OI, athletics coaches should be cautious with high training volume during conditioning phases and training camps. Future research should focus on identifying risk factors for OI to lower the overall and monthly injury incidence rates and injury incidence rate per 1000 athletics-hours of training to increase athlete availability in elite athletics.

## Data Availability

The datasets used and/or analyzed during the current study are available from the corresponding author on reasonable request.

## Abbreviations

BMI: Body mass index
CI: Confidence Interval
M/L: Middle- and long-distance runner
OI: Overuse injuries
SD: Standard deviation
